# Retinal changes of primary vitreoretinal lymphoma after intravitreal methotrexate

**DOI:** 10.1186/s12886-022-02604-7

**Published:** 2022-09-20

**Authors:** Tingting Jiang, Junxiang Gu, Shixue Liu, Qing Chang

**Affiliations:** 1 Department of Ophthalmology, Eye and ENT Hospital, Fudan University, 83 Fenyang Road, Shanghai, 200031 China; 2 Shanghai Key Laboratory of Visual Impairment and Restoration, Shanghai, 200031 China; 3 Key Laboratory of Myopia of National Health Commission, Fudan University, Shanghai, 200031 China; 4 Key Laboratory of Myopia, Chinese Academy of Medical Sciences, Shanghai, 200031 China

**Keywords:** Primary vitreoretinal lymphoma, Methotrexate, Optical coherence tomography, Intravitreal chemotherapy

## Abstract

**Background:**

To identify retinal changes using spectral-domain optical coherence tomography (SD-OCT) and ultra-widefield images in eyes with primary vitreoretinal lymphoma (PVRL) during intravitreal methotrexate (MTX) treatment.

**Methods:**

This study retrospectively reviewed 111 eyes of 58 patients with vitreous cytology-proven confirmed PVRL, who received intravitreal injections of MTX.

**Results:**

At the initial visit, the OCT manifestations included vitreous cells (105 eyes, 94.6%), intraretinal infiltration (44 eyes,39.6%), subretinal infiltration (45 eyes, 40.5%,), retinal pigment epithelium (RPE) abnormalities (66 eyes, 59.5%), disruption of the ellipsoid zone (58 eyes, 52.3%), subretinal fluid (4 eyes, 3.6%), RPE detachment (PED) (28 eyes, 25.2%), epiretinal membrane (ERM) (8 eyes, 7.2%), macular edema (10 eyes, 9%). After therapy, tumor regression was achieved in all eyes. Between the initial presentation and regression, the vitreous cells (94.6% vs. 0%, *P* < 0.001), intraretinal infiltration (39.6% vs. 0%, *P* < 0.001), RPE abnormalities (59.5% vs.19.8%, *P* < 0.001), PED (25.2% vs.0%, *P* < 0.001), and subretinal infiltration (40.5%vs.16.2%, *P* < 0.001) were significantly reduced. The fundus photography findings all improved after therapy. The mean Logarithm of the Minimum Angle of Resolution (logMAR) for the best corrected visual acuity (BCVA) at presentation was 0.79 ± 0.81 (range, 0–2.9), which improved to 0.70 ± 0.97 (range, 0–2.9, *P* = 0.01) at the final visit.

**Conclusions:**

SD-OCT combined with ultra-widefield imaging, which can reflect retinal changes, are valuable tools for monitoring the effect of PVRL treatment.

## Background

Primary vitreoretinal lymphoma (PVRL) is a subgroup of primary central nervous system lymphoma (PCNSL) that affects the retina and vitreous or optic nerve [1, 2]. The incidence of this disease is approximately 0.047 cases per 100,000 people per year based on data from North America [3]. Most cases are classified as diffuse large B-cell lymphoma and most patients (56%–90%) with PVRL develop central nervous system (CNS) involvement [4]. Early diagnosis is difficult because PVRL often presents with various ocular manifestations. It frequently masquerades as posterior uveitis, and the initial response to steroids can further mask the disease. Cytological examination of vitreous samples remains the gold standard for the definite diagnosis of PVRL [1, 5, 6]. Ancillary tests for the diagnosis of PVRL include a high level of interleukin (IL)-10 in the intraocular fluid with an IL-10/IL-6 ratio greater than 1.0, polymerase chain reaction (PCR) for immunoglobulin gene rearrangements, MYD88 mutational testing, and bespoke next-generation sequencing panels [6–9].

PVRL is often associated with PCNSL and is a potentially fatal intraocular malignancy. With the advent of more therapies such as intraocular chemotherapy and autologous stem cell transplantation, the prognosis of the disease is expected to improve. At present, the most commonly used drug for intraocular chemotherapy is methotrexate (MTX) [10], an anti-metabolite that was first used to treat PVRL in the mid-1990s [11]. Intravitreal injection of MTX has been reported to be effective in achieving intraocular tumor remissions with acceptable side effects [10–26].

However, a wide range of injection schedules have been described without a consensus on the ideal guideline. Moreover, since intraocular chemotherapy is associated with complications such as corneal epitheliopathy, cataract, and optic neuropathy [21], it is better to reduce the injection frequency if possible. Therefore, it is important to identify reliable indicators for evaluating the treatment effect and determining the injection intervals and duration. Optical coherence tomography (OCT) and the ratio of IL-10 and IL-6 in the intraocular fluid have been reported as objective and repeatable methods to assess the efficacy of treatment [12–17, 27]. Although several reports have described OCT findings for PVRL [28–35], the sample sizes in most of them were small and the duration of follow-up was short.

In this study, we summarized the OCT features in a relatively large number of PVRL patients (111 eyes in 58 patients), who were treated with intravitreal injections of MTX. Ultra-widefield images and the IL-10 and IL-6 concentrations in the anterior chamber humor during treatment were also evaluated.

## Material and methods

### Ethics

In this retrospective study, the data for 58 patients (111 eyes) who were diagnosed with PVRL at the Eye and ENT (Ear Nose Throat) Hospital of Fudan University in Shanghai, China from 2012 to 2021 were reviewed. The ethics committee of the Hospital approved the study, which was performed in accordance with the tenets of the declaration of Helsinki.

### Patients

The inclusion criteria were as follows: 1) confirmed diagnosis of PVRL based on the cytology of the vitreous biopsy. The typical lymphoma cells are large B-cell lymphoid cells with a scanty basophilic cytoplasm, an increased nucleus:cytoplasm ratio, with hypersegmented, multivariable-shaped nuclei, multiple nucleoli, and a coarse chromatin pattern [36]. 2) Ultra-widefield imaging, including wide-field fundus photography and wide-field fundus autofluorescence (FAF), IL-10 and IL-6 concentrations in the anterior chamber fluid, and OCT images were available for analysis at both the initial and follow-up visits; and 3) patients received intravitreal injections of MTX. The demographic information, ophthalmologic records, and history of CNS lymphoma were retrospectively reviewed. Patients who were lost to follow-up (5 patients) and those who had OCT images with quality that was too low for analysis (3 patients) were excluded.

### Ophthalmic tests

All patients underwent spectral domain OCT (SD-OCT; Spectralis HRA + OCT; Heidelberg Engineering, Heidelberg, Germany) using a 30° volume scan pattern with 25 horizontal linear B-scans positioned at the center of the fovea.

**Fig. 1:**
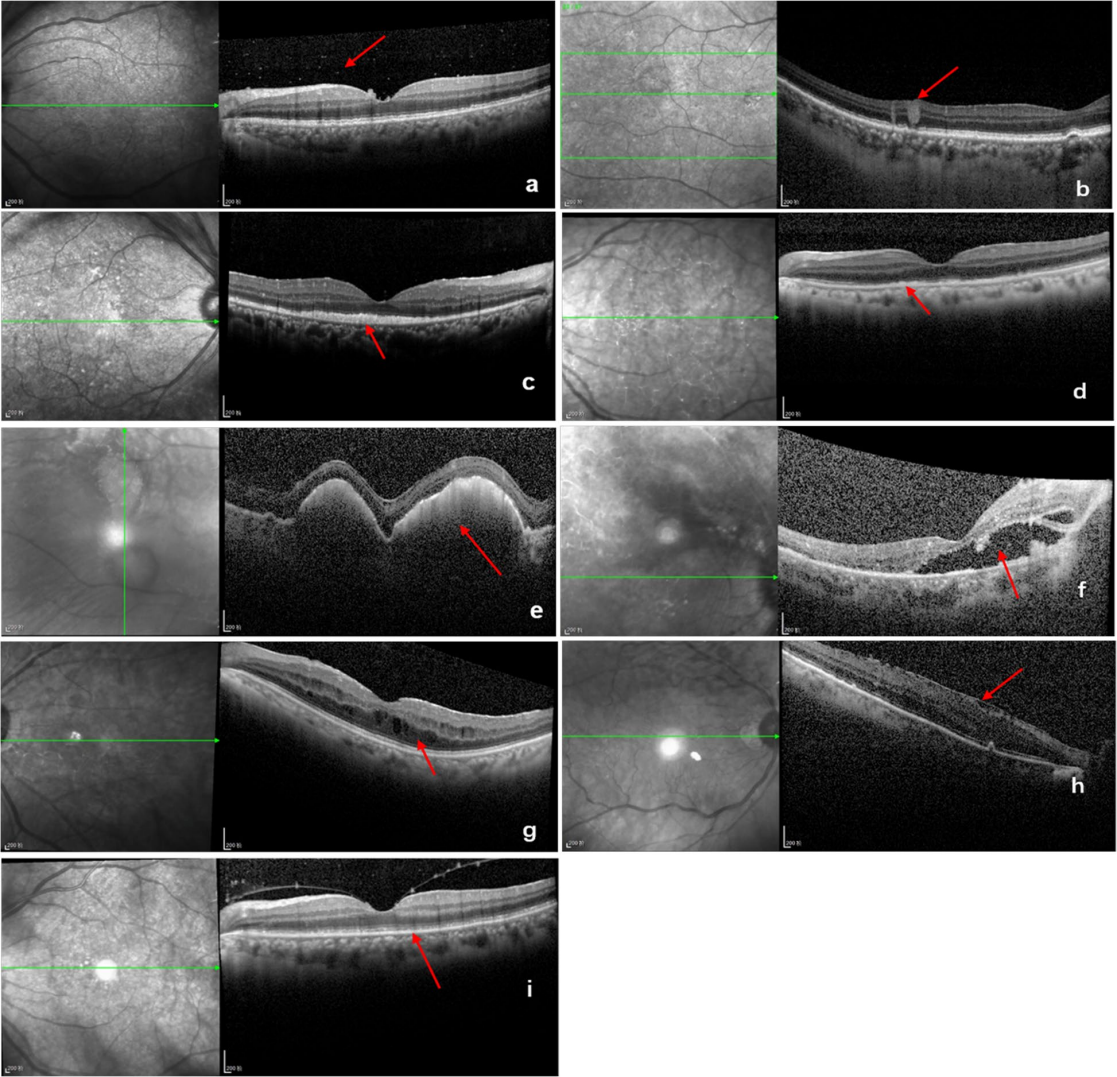
Representative SD-OCT images displaying characteristic features of PVRL. **a** vitreous opacities (arrow) **b** intraretinal infiltration (arrow) **c** subretinal infiltration (arrow) **d** retinal pigment epithelium (RPE) abnormalities (hyperreflective nodules or irregularity) (arrow) **e** RPE detachment (arrow) **f** subretinal fluid (arrow) **g** macular edema (arrow) **h** epiretinal membrane (arrow) **i** disruption of the ellipsoid zone (arrow)

SD-OCT images were used to evaluate the following features: vitreous cells (dense particles in the posterior vitreous) (Fig. 1a), intraretinal infiltration (hyperreflective lesions within the neuroretina), (Fig. 1b), subretinal infiltration (hyperreflective lesions between neurosensory retina and RPE), (Fig. 1c), retinal pigment epithelium (RPE) abnormalities (irregularity and hyperreflective nodules of the RPE) (Fig. 1d), RPE detachment (PED) (nodular deposits between the RPE and Bruch membrane) (Fig. 1e), subretinal fluid (Fig. 1f), macular edema (Fig. 1g), epiretinal membrane (ERM) (Fig. 1h), disruption of the ellipsoid zone (Fig. 1i). All SD-OCT images were analyzed by two independent observers blind to the experimental conditions. Ultra-widefield fundus photography and FAF imaging were performed. Complete remission was defined as the absence of cells in the vitreous and the resolution of retinal or RPE infiltrates. Relapse was defined as the re-appearance of vitritis and retinal or RPE infiltrates [37].

### Determination of IL-10 and IL-6 levels

The aqueous humor was collected from each patient and the levels of IL-10 and IL-6 in the aqueous humor were determined using a standard enzyme-linked immunoassay according the manufacturer’s instructions. A cutoff of 30 pg/mL IL10 in the aqueous humor was used in our study [38].

### Treatment

All patients were treated with systemic chemotherapy, radiotherapy, and intraocular chemotherapy individually or in combination. Intravitreal MTX (400 μg in 0.1 ml) was injected in three phases: induction phase (twice weekly for 2 weeks), consolidation phase (weekly for 1 month), and maintenance phase (monthly for 2–10 months). During the follow-up period, the disease activity was assessed. If complete remission was achieved and the IL-10 concentration in the aqueous humor reached an undetectable level, the intravitreal injection of MTX was terminated. In the event of relapse, MTX was re-started.

### Statistical analysis

Statistical analysis was performed using SPSS (Version 20, SPSS, Inc, Chicago, IL). SD-OCT features at the initial presentation and regression were compared using the chi-squared test or Fisher’s exact test. A Wilcoxon signed rank test was used to compare the differences in the visual acuities before and after treatment. A p value less than 0.05 was considered statistically significant.

## Results

A total of 111 eyes in 58 patients with cytological-proven PVRL were enrolled in this study. All patients were diagnosed with diffuse large B-cell lymphoma by vitreous biopsy. The baseline demographics, clinical findings, and treatment are summarized in Table 1. The average age was 56.59 ± 12.17 years (range, 32–86 years). Of the 58 patients, 53 patients (91.4%) had bilateral involvement. The mean follow-up period was 3.69 ± 2.33 years (range, 1–9.9 years). CNS lymphoma occurred in 38 (65.5%) patients.

**Table 1:** Baseline characteristics, clinical, and treatment features of patients with PVRL treated with intravitreal MTX

Demographics, Ocular Features, and Treatment	*N* = 58 Patients (%)
	*n* = 111 eyes (%)
Sex	
Male	21 (36.2%)
Female	37 (63.8%)
Mean age at presentation (years)	56.6 ± 12.2 (range, 32–86)
Central nervous system lymphoma	38 (65.5%)
Follow-up (years)	3.7 ± 2.3 (range, 1–9.9)
Onset of ocular symptoms to diagnosis (months)	15.8 ± 14.2 (range, 1–53.2)
Laterality	
Unilateral	5 (8.6%)
Bilateral	53(91.4%)
Treatment	
Systemic chemotherapy	53 (91.4%)
Intravitreal chemotherapy	111 eyes (100%)
External beam radiotherapy	15 (25.9%)
Recurrence	18 eyes (16.2%)
Outcome (at the final visit)	
Regression	100%

*PVRL* Primary vitreoretinal lymphoma, *MTX* Methotrexate

The SD-OCT features of the 111 eyes at initial presentation are summarized in Table 2. These SD-OCT features included vitreous cells (105 eyes, 94.6%), intraretinal infiltration (44 eyes,39.6%), subretinal infiltration (45 eyes, 40.5%), RPE abnormalities including hyperreflective nodules or irregularity (66 eyes, 59.5%), disruption of the ellipsoid zone (58 eyes, 52.3%), subretinal fluid (4 eyes,3.6%), PED (28 eyes, 25.2%), ERM (8 eyes, 7.2%), macular edema (10 eyes, 9%). In addition, retinal edema, subretinal fluid, and retinal full-thickness infiltration commonly occurred in patients with a chronic course.

**Table 2:** OCT findings for patients with PVRL

OCT feature	At first visit *n* = 111 eyes (%)	At last visit *n* = 111 eyes (%)	*P* value
Vitreous cells	105 (94.6%)	0%	*P* < 0.001
Intraretinal infiltration	44 (39.6%)	0%	*P* < 0.001
Subretinal infiltration	45(40.5%)	18 (16.2%)	*P* < 0.001
RPE abnormalities (hyperreflective nodules or irregularity)	66 (59.5%)	22 (19.8%)	*P* < 0.001
Disruption of the ellipsoid zone			
Subretinal fluid	58(52.3%)	52 (46.8%)	
RPE detachment	4 (3.6%)	0%	
ERM	28 (25.2%)	0%	*P* < 0.001
Macular edema	8 (7.2%)	8 (7.2%)	
	10 (9.0%)	0%	

*OCT* Optical coherence tomography, *RPE* Retinal pigment epithelium, *ERM* Epiretinal membrane, *PVRL* Primary vitreoretinal lymphoma

**Fig. 2:**
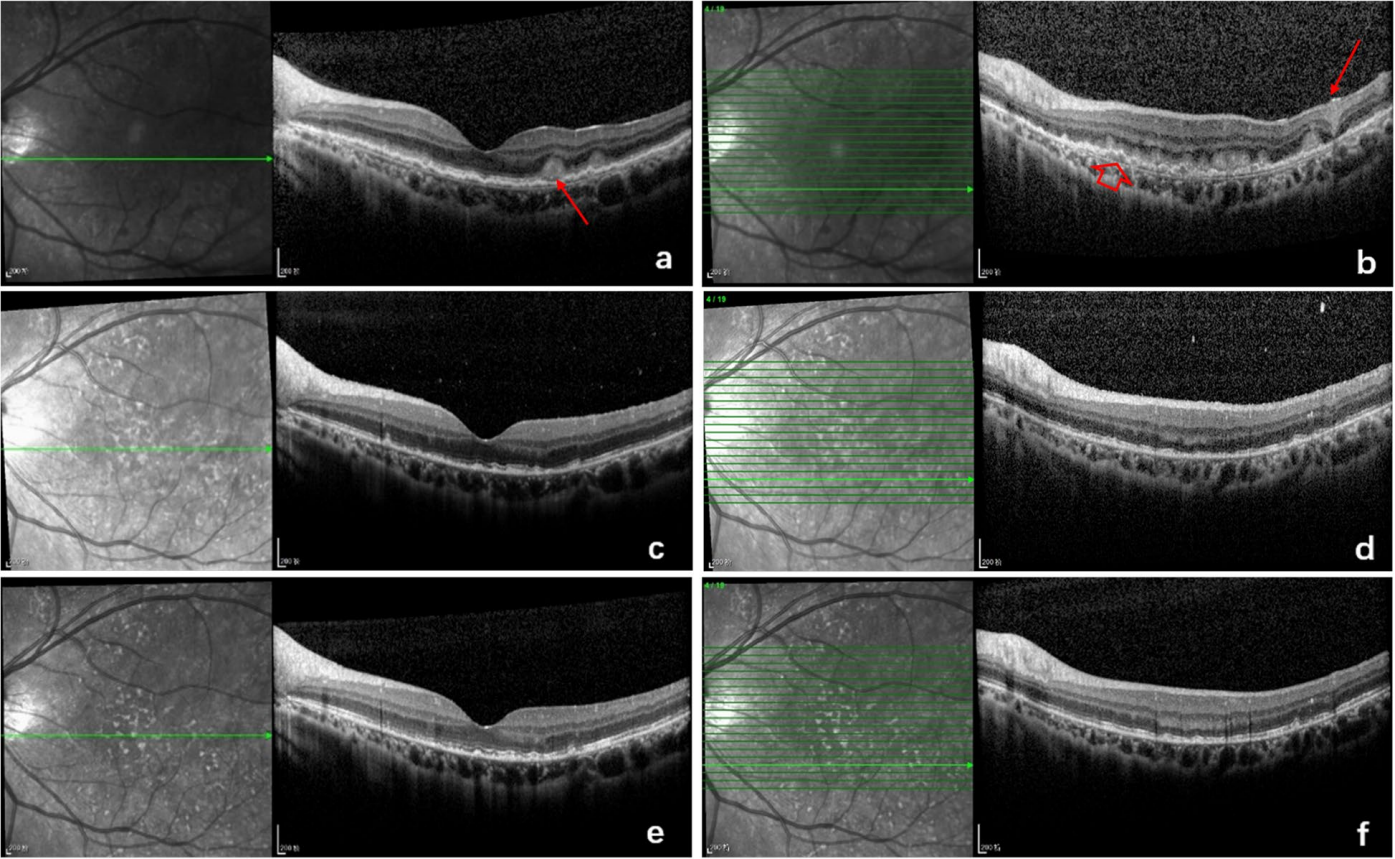
Serial SD-OCT images of a case. **a** and **b** Initial presentation revealed subretinal infiltration (arrow), intraretinal infiltration (arrow), and RPE detachment (hollow arrow). **c** and **d** After consolidation treatment, intraretinal infiltration and subretinal infiltration disappeared. RPE detachment subsided. Disruption of the ellipsoid zone and RPE hyperreflective nodules were revealed. **e** and **f** Two months after maintenance treatment, RPE hyperreflective nodules further decreased and the disruption of the ellipsoid zone improved

**Fig. 3:**
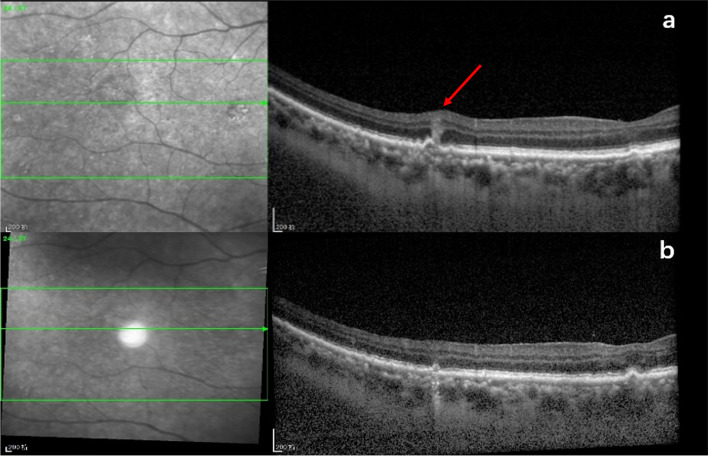
Serial SD-OCT images of a case. **a** Initial presentation revealed a vertical hyperreflective lesion (VHRL) connected with the PED (arrow). **b** After induction treatment, the vertical hyperreflective lesion (VHRL) subsided and the PED improved

The treatment modality for PVRL was systemic chemotherapy (91.4%), intravitreal chemotherapy (100%), or brain radiotherapy (25.9%). Patients with PCNSL or bilateral involvement received systemic chemotherapy combined with intraocular chemotherapy, some patients received brain radiation therapy simultaneously. All of the eyes responded to the intravitreal injections. At the end of induction treatment, all the abnormalities alleviated. At the end of consolidation phase of treatment, vitreous cells (94.6% vs. 24.3%, *P* < 0.001), intraretinal infiltration (39.6% vs. 22.5%, *P* < 0.01), subretinal infiltration (40.5%vs 27%, *P* < 0.05), RPE abnormalities (59.5% vs.45%, *P* < 0.05), PED (25.2% vs.11.7% *P* < 0.05), subretinal fluid (3.6% vs.0%), macular edema (9% vs.5.4%) further improved (Figs. 2 and 3). However, recurrence was observed in 18 eyes (16.2%). Six of them(5.4%)did not follow the treatment regimen and didn’t receive an adequate number of injections at the initial stage. Of the 18 eyes with tumor recurrence, OCT images were characterized by subretinal infiltration, abnormal RPE, and intraretinal infiltration. For these eyes, intravitreal MTX injections were re-started on a weekly basis. By the last visit, complete regression had been achieved in all eyes. The median number of intravitreal injections was 12 (range, 10–18). Vitreous cells (94.6% vs. 0%, *P* < 0.001), intraretinal infiltration (39.6% vs. 0%, *P* < 0.001), RPE abnormalities (59.5% vs.19.8%, *P* < 0.001), PED (25.2%vs.0%, *P* < 0.001), and subretinal infiltration (40.5%vs.16.2% %, *P* < 0.001) were significantly reduced at regression compared to the findings at the initial presentation. At the last visit, the disruption of the ellipsoid zone (52 eyes, 46.8%), subretinal fibrosis (18 eyes, 16.2%), RPE abnormalities (22 eyes, 19.8%), and the macular membrane (8 eyes, 7.2%) were noted. The mean Logarithm of the Minimum Angle of Resolution (logMAR) for the best corrected visual acuity (BCVA) at presentation was 0.79 ± 0.81 (range, 0–2.9), and the final logMAR BCVA improved to 0.70 ± 0.97 (range, 0–2.9, *P* = 0.01).

**Fig. 4:**
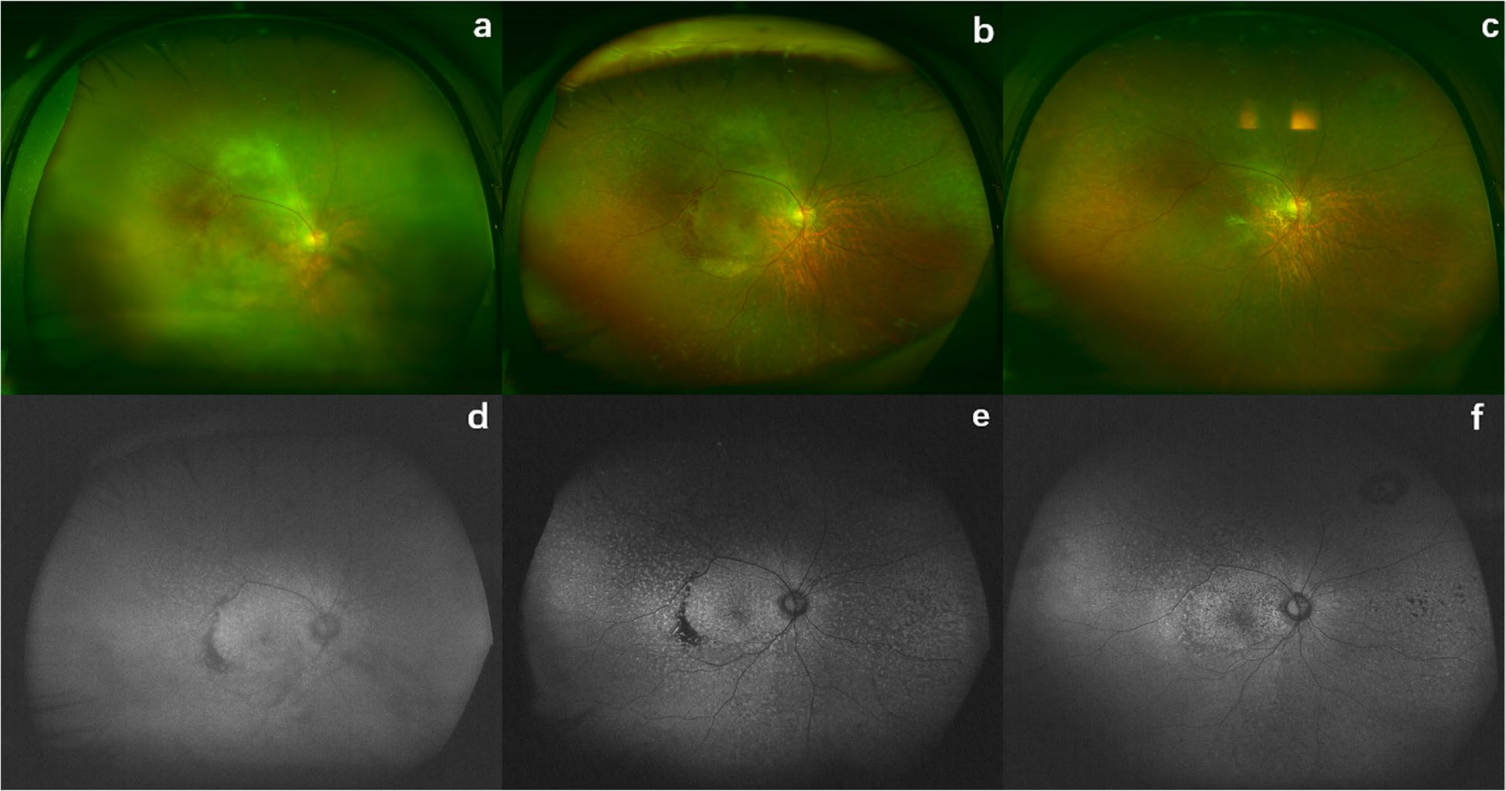
Images from a case. At presentation, **a** ultra-widefield fundus photography of the right eye demonstrated vitritis, retinal hemorrhage, and subretinal lesions. **b** After induction treatment, the vitritis and subretinal lesions improved. There was retinal hemorrhage and RPE pigment changes. **c** After consolidation treatment, the vitritis, retinal hemorrhage and subretinal lesions disappeared. **d** At presentation, fundus autofluorescence shows hyperfluorescence corresponding to the lesions and hypofluorescence due to retinal hemorrhage. **e** After induction treatment, a mixture of granular hyperfluorescence and hypofluorescence was revealed in the fundus autofluorescence including fluorescence blockage by retinal hemorrhage. **f** After consolidation treatment, a hypoautofluorescent and hyperautofluorescent mottled pattern was observed in the fundus autofluorescence

Wide-field fundus photography findings included vitritis (105 eyes, 94.6%), optic nerve infiltration (7 eyes,6.3%), retinal hemorrhage (10 eyes, 9%), retinal infiltration (44 eyes,39.6%) and sub–retinal lesions (45 eyes, 40.5%) at the initial presentation, and were all improved after therapy (Fig. 4). Before treatment, FAF results showed a hypoautofluorescent and hyperautofluorescent mottled pattern in 47 eyes (42.3%). Hyperfluorescence occurred in 21 eyes (18.9%) (Fig. 4). Unremarkable FAF was found in 43 eyes (38.7%). Resolution was observed after treatment leading to hypo-AF.

The IL-10/IL-6 ratio at baseline was greater than 1.0 in all the eyes. After therapy, the ratio decreased significantly. However, the IL-10 concentration increased again in recurrent cases. At the last visit, the IL-10 value was undetectable in all the eyes.

During intraocular chemotherapy, 60 eyes (54.1%, 60/111) developed corneal epithelial lesions, which were improved after reduction of the injection frequency and local eyedrops treatment. Transient intraocular pressure increased in 4 eyes (3.6%, 4/111), which returned to normal after local eyedrops were administered. No drug resistance was found.

## Discussion

Currently, there is limited evidence to guide the management of PVRL. Treatment regimens include radiation, systemic chemotherapy, intraocular chemotherapy, and autologous stem cell transplantation. Combination therapy is often required for PVRL treatment and ophthalmologists have the responsibility to initiate intraocular chemotherapy. Intraocular injection of MTX is used widely with confirmed effectiveness for the treatment of PVRL [10–26]. However, the treatment schedules varies, and thus it is important to establish a treatment protocol for PVRL based on the therapeutic response. Different parameters are used to measure the response to the therapy. OCT, which is superior to BCVA, is considered a useful tool to monitor the responsiveness of PVRL to treatment and can assist in the management of the disease [12, 13, 27, 32, 33]. In this study, besides OCT, we also reported the changes of wide-angle fundus photography and interleukin levels during treatment, which provided a comprehensive illustration of the intraocular condition in PVRL patients. We found that SD-OCT, as well as ultra-widefield images and the IL-10 and IL-6 levels in the intraocular fluid, is favorable for the establishment of PVRL treatment guidelines.

In our patients, besides vitreous opacities, intraretinal infiltration, subretinal infiltration, RPE abnormalities, disruption of the ellipsoid zone, and PED were common features in eyes with PVRL at the initial visit, which is consistent with other reports [5, 12, 13, 28–33, 35]. Among them, lesions in the outer retina and RPE level accounted for a high proportion. These manifestations improved after treatment. At the final visit, most of these features were significantly reduced. The disruption of the ellipsoid zone remained the most frequent morphologic characteristic after therapy. This may be due to lymphomatous infiltration into the RPE, leading to irreversible damage to the photoreceptor outer segment [12, 33]. The proportions of OCT characteristics varied between studies, which may be due to different sample sizes and the diversity of OCT manifestations in PVRL patients.

The source of lymphomatous cells within the retina is still unclear. Chan et al. demonstrated that lymphoma cells can migrate from the vitreous into the retina in a murine model of primary intraocular lymphoma [37]. A clinical study by Yang et al. showed that lymphoma cells appeared initially in the vitreous or the sub-RPE space and subsequently invaded into the neurosensory retina or subretinal space [13]. Deák et al. found that lymphoma cells may enter the retina through retinal blood vessels and cross the retina to reach the sub-RPE space based on vertical hyperreflective lesions (VHRLs) on OCT [39]. However, not every sub-RPE infiltrate is associated with the previous VHRLs on OCT. In our study, VHRLs often appeared in previous irregular RPE areas during progression and were associated with PED. In patients with a chronic disease course, retinal edema, subretinal fluid, and full-layer infiltration were more common.

Besides OCT, we found that wide-field images were also valuable to evaluate the changes in eyes with PVRL in response to the treatment, since many lesions were localized in the periphery. In addition, retinal and RPE infiltrates were sometimes more noticeable on FAF than on color fundus photography. In our study, wide -field fundus photography findings were significantly improved after therapy. The most common manifestation of FAF was a hypoautofluorescent and hyperautofluorescent mottled pattern (42.3%).

Hypoautofluorescence indicates the presence of intraretinal or subretinal lymphomatous infiltration or RPE atrophy, whereas hyperautofluorescence is correlated with the impairment of lipofuscin metabolism at the RPE level [40, 41]. Areas of hypo-AF relate to coalescing RPE holes with surrounding island of hyper-AF corresponding to scrolled RPE have been reported [34]. These manifestations should be distinguished from areas of disease activity to prevent over-treatment.

In the present study, the IL-10 and IL-6 levels changed in parallel with the response to treatment. IL-10 levels were significantly lower than baseline two weeks after intraocular injection, and they keep decreasing with treatment. The levels of IL-10 and the ratios of IL-10/IL-6 were elevated in patients who relapsed. These findings suggested that analysis of the cytokines in the aqueous humor is also helpful to evaluate the effect of treatment and to detect clinical conditions such as recurrence, which is consistent with previous reports [14–17]. In this study, IL-10 concentration was not detectable after an average of 6.4 months of injection.

Although intravitreal MTX has been used for the treatment of PVRL, the number of intravitreal injections varies greatly, ranging from 2 to 26 [10, 12–16, 19–26]. In our study, intravitreal MTX was used in three phases: induction (twice weekly for 2 weeks), consolidation (weekly for 1 month), and maintenance (monthly for 2–10 months). The mean injection number was 12, ranging from 10 to 18. Complete regression was achieved in all eyes along with improved BCVA, suggesting that the injection protocol was effective based on OCT, wide-angle images, and cytokine tests.

Recurrence was found in 18 eyes (16.2%). OCT revealed that subretinal infiltration, intraretinal infiltration and RPE abnormalities occurred more often than other changes, suggesting that these changes may be early indicators of disease recurrence. These findings are consistent with the results from previous studies [13, 32].

The limitations of our study include its retrospective nature; thus, future prospective studies are required to confirm the findings of this study. In addition, SD-OCT is difficult to perform in eyes with severe vitreous haze. Those who had OCT images with quality that was too low for analysis were excluded. Considering the limited number of patients, the data will serve as a reference in clinical practice and for the overall improvement of PVRL therapy. Further studies with larger sample sizes are needed. However, the strength of our study is the availability of serial OCT images for the patients during treatment. Besides the OCT features, ultra-widefield images and the levels of IL-10 and IL-6 in the anterior chamber humor were also evaluated. The above combination is more accurate than OCT alone for formulating a treatment plan. Moreover, the patients were followed up for long periods.

## Conclusions

In conclusion, we found OCT changes in eyes with PVRL during treatment, suggesting that OCT is a valuable tool for monitoring the treatment response to intraocular chemotherapy. Additionally, wide-field imaging and the IL-10 and IL-6 concentrations in the intraocular fluid are also useful for evaluation. Individualized intraocular chemotherapy schedules may be established through comprehensive assessments based on OCT, wide-field imaging, and the IL-10 and IL-6 concentrations in the intraocular fluid.

## Data Availability

The datasets used and/or analysed during the current study available from the corresponding author on reasonable request.
